# Analysis of Expression of Programmed Cell Death 1 Ligand 1 (PD-L1) in Malignant Pleural Mesothelioma (MPM)

**DOI:** 10.1371/journal.pone.0121071

**Published:** 2015-03-16

**Authors:** Susana Cedrés, Santiago Ponce-Aix, Jon Zugazagoitia, Irene Sansano, Ana Enguita, Alejandro Navarro-Mendivil, Alex Martinez-Marti, Pablo Martinez, Enriqueta Felip

**Affiliations:** 1 Medical Oncology Service/Vall d´Hebron Institute Oncology, Vall d’Hebron University Hospital, Barcelona, Spain; 2 Medical Oncology Service/ 12 de Octubre University Hospital, Madrid, Spain; 3 Pathology Department/ Vall d’Hebron University Hospital, Barcelona, Spain; 4 Pathology Department/12 de Octubre University Hospital, Madrid, Spain; University of Pittsburgh, UNITED STATES

## Abstract

**Background:**

The increasing incidence and poor outcome associated with MPM requires finding effective treatment for this disease. PD1/PD-L1 pathway plays a central role in tumor immune evasion and appears to be predictive and prognostic marker. PD-L1 is expressed in many different human cancers but its role in MPM has yet to be established. The aim of this study is to evaluate the expression of PD-L1 in MPM.

**Methods:**

119 MPM patients (p) from two institutions between November 2002 and February 2014 were reviewed. Formalin-fixed, paraffin-embedded tissue was stained with anti-PD-L1 (clone E1L3N). Cases showing more than 1% of tumor cells expression of PD-L1 were considered positive.

**Results:**

PD-L1 was analyzed in 77 p with tumor tissue available and was positive in 20.7% p (14 samples in membrane, 16 in cytoplasm and 4 in immune infiltrate). PD-L1 intensity was weak in 56.2%, moderate in 25% and strong in 18.7% p. There was a significant relationship between PD-L1 expression and histology (PD-L1 expression 37.5% in no-epithelioid tumor and 13.2% in epithelioid; p=0.033). The median survival in p PD-L1 positive was 4.79 vs 16.3 months in p PD-L1 negative (p=0.012).

**Conclusions:**

We have shown PD-L1 is expressed in 20% of patients, associated with no epithelioid histology and poor prognostic in MPM. Our results suggest PD-L1 warrants further exploration in selecting p for immunotherapy.

## Introduction

Malignant pleural mesothelioma (MPM) is a locally aggressive malignancy arising from the mesothelial cells lining the pleura with a median survival time for untreated patients ranging from 4 to 12 months [1]. The current standard for systemic treatment of advanced MPM is the combination of chemotherapy with cisplatin and folate drugs analogs, but treatment results in an improvement in median survival of less than 3 months [2,3]. Multimodality therapy with chemotherapy, surgery and radiation therapy has been shown to potentially improve survival in a highly selected group of patients with MPM [4,5].

The increasing incidence and poor outcome associated with MPM require urgently novel therapeutic strategies to improve the prognosis. There is some evidence that MPM is an immunogenic tumor that induces immune recognition, infiltration of immune cells and death mediated by autoimmunity [6–8]. Clinical studies have provided evidence that lymphocyte invasion influences prognosis in MPM [7,9]. Additionally MPM seems to be responsive to immunotherapy and there are some cases of spontaneous regression reported suggesting antitumor immune response [10–12].

The programmed cell death (PD-1/PD-L1) pathway plays a critical role in to limit the activity of T cells in peripheral tissues at the time of an inflammatory response to infection and to limit autoimmunity. In tumors this pathway controls the tumor immune escape. PD-1 receptor is a negative regulator of T-lymphocyte and acts as a coinhibitory receptor to prevent off target immune activation [13]. PD-1 binds to programmed cell death ligand 1 (PD-L1, B7-H1), the predominant mediator of immunosuppression. PD-L1 is an immunomodulatory cell—surface glycoprotein that is primarily expressed by antigen-presenting cells on myeloid dendritic cells, activated T cells and some nonhematopoietic tissues. PD-L1 serves to regulate the cellular immune response [14]. Binding of PD-L1 to its receptor PD-1 inhibits proliferation of activated T cells in peripheral tissues leading to “T-cell exhaustion”, a T cell hyporeactive condition [15].

It has been reported that PD-L1 is broadly expressed in several malignant tumors including carcinomas of the esophagus, kidney, lung cancer and brain tumors among others [16–21]. Moreover, the expression levels of these molecules have been shown to correlate with the prognosis of the patients in some cases [16–19,22]. However, most of the studies have been conducted on frozen specimens because of the lack of an appropriate anti human PD-L1 antibody that can stain PD-L1 on formalin fixed paraffin embedded (FFPE).

Newly developed immune checkpoint inhibitors have shown promising results in phase I trials in several tumor types [23–25]. Preliminary evidence of these trials suggests that tumor expression of PD-L1 by immunohistochemistry (IHC) is a promising predictive biomarker of response to anti-PD-1/PD-L1. However, there is no consensus about the definition of positivity of PD-L1. Distinct PD-L1 antibodies have been developed to each agent (nivolumab, pembrolizumab and MPDL3280A), each with its own technical specifications and definition of positivity [23,25,26].

There are limited data on the prevalence and the prognostic role of PD-L1 expression in MPM. A mouse model reported that PD-L1 is highly expressed in the tumor cells and within tumor stroma and PD-L1 blockade results in T cell activation [27]. A recent clinical study showed PD-L1 is expressed in 40% of patients in a series of MPM using 5H1 antibody [28].

To further explore the prevalence and roles of PD-L1 in MPM, we measured the levels of PD-L1 protein using E1L3N antibody in 119 samples from two retrospective MPM cohorts. The aim of this study is to investigate the association between PD-L1 and clinicopathological parameters in MPM and the potential association with prognosis.

## Methods

### Study population

One hundred nineteen consecutive cases of MPM were collected from January 2000 to April 2014 at Vall d´Hebron University Hospital and 12 de Octubre University Hospital. All patients presented histologically proven diagnosis of malignant pleural mesothelioma. Clinicopathologic information gathered included complete history, age, sex, performance status (PS), asbestos exposure, tumor stage and histology subtype. The tumor stage was defined according to the International Union Againts Cancer´s tumor-node-metastasis 7^th^ classification and sub-classified histologically according to WHO guidelines [29].

### IHC analysis

Tissue specimens were obtained from the primary mesothelioma at the time of diagnosis. No patients received prior neoadjuvant therapy. Cases included epithelioid, sarcomatoid and biphasic subtypes of malignant mesothelioma. All of the tumor samples from the 119 patients were obtained from Department of Pathology. The same method was used for each patient. Approval for the use of the tissue used in research was obtained from the ethical local committee from both hospitals and all patients gave written inform consent before enrollment. Each sample was assessed histologically for tumor tissue by two pathologists (IS and AA)

Immunohistochemistry analysis was carried out in sections that were deparaffinied (EZprepTMx10) in an oven for 30 minutes at 60ºC followed by three serial xylenes incubations. Sections were then rehydrated in grades alcohols and subjected to antigen retrieval using XS Tris Buffered Saline with Tween 20 and boiled for 20 minutes. IHC using rabbit monoclonal primary PD-L1 antibody (cloneE1L3N)XP Cell Signaling at 1:1200 dilution) was carried out using 4 mm-thick FFPE tissue sections on a Benchmark XT autostainer (Ventana Medical System) with standard antigen retrieval methods. The SignalStain DAB substrate kit (#8959) was used according to the manufacturer´s instructions. Human placenta was included as positive control for endogenous PD-L1. The antibodies used for tumor-infiltrating lymphocytes (TILs) were anti-human CD4 (clone BC/1F6;Abcam) and anti-human CD8 (clone 4B11;Novocastra). For anti-human CD8 deparaffinized sections were immersed into 10mmol/L of pretreated citrate buffer (pH6.0), incubated at 95ºC for 20 minutes, and allowed to cool to room temperature. For anti-human CD4, pretreated Tris-EDTA buffer (pH0.9) was used for antigen retrieval at 95º for 20 minutes.

Before scoring, specimens with no tumor cells or questionable cells from inflammatory cells were excluded from analysis. All IHC stained sections were initially evaluated and scored by two pathologist and discrepancies in interpretation of scoring were resolved by consensus. Tumors with ≥1% of tumor cells stained either in membrane or cytoplasm were considered positive for PD-L1. The expression of PD-L1 was evaluated according to the intensity of the staining and scored in a furthered system as follows: 0, negative;1, weak expression;2, moderate expression but weaker than placenta; and 3, equivalent or stronger expression than placenta. To examine TILs, the number of cells per microscopic with immunoreactivity to CD4 and CD8 were counted and we defined the percentage average media in the slide as the number of TILs for each case.

## Statistical

Data were censored at last follow up for patients without relapse or death. Associations of PD-L1 expression with clinicopathologic features were evaluated with Fisher exact tests and Wilcoxon Mann-Whitney tests. Overall survival was calculated from diagnosis of malignancy until death due to any cause or until the date of last follow-up visit for still alive patients. Survival analysis that compared PD-L1 expressing tumors was carried out using the Kaplan-Meier curves and the significance was verified by a log-rank test. All p values were determined by two-sided tests and p values <0.05 were considered significant. Multivariate analysis was done using the Cox regression model including only the clinical variables and antibody expression markers that showed significance in univariate analysis. Data analysis and summary graphs were produced by the R statistical software version 3.0.1.

## Results

### Patient population

We studied 119 patients with MPM whose clinicopathologic characteristics are summarized in Table 1. The total sample comprised 78 epitheliod, 12 sarcomatoid, 5 biphasic and 24 cases with histology type not specified of MPM. The median age was 69 years (range 42–90). Patients were predominantly male (71.4%), smokers (50.5%) and had previous asbestos exposure (44.5%). Out of the entire group, none of the patients was considered for extrapleural pneumonectomy and 78 (65.5%) patients were treated with chemotherapy. Platinum plus pemetrexed was used in 89% of patients and platinum plus gemcitabine in 8%.

**Table 1:** Baseline patients characteristics.

Clinical characteristics		N	%
Gender	Male	85	71.4
	Female	34	28.6
Smoke	Current	33	27.7
	Former	28	23.5
	No smoker	41	34.4
	Unknown	17	14.3
Asbestos exposure	Yes	53	44.5
	No	38	31.9
	ND	28	23.5
PS	0	19	20.2
	1	61	64.9
	2	10	10.6
	3	4	4.3
	ND	25	26.6
Localization	Right	64	53.8
	Left	45	37.8
	ND	10	7.4
Pleural efussion	Yes	92	77.3
	No	14	11.8
	ND	13	10.9
NLR*	≥5	30	27.5
	<5	79	72.4
Stage	I-II	27	22.6
	III-IV	77	64.7
	ND	15	12.6
Histology	Epithelioid	78	65.5
	Sarcomatoid	12	10.1
	Biphasic	5	4.2
	No specify	24	20.2
Systemic treatment	Yes	78	65.6
	No	31	26.1
	ND	10	8.4

NLR*: neutrophil to lymphocyte ratio; ND: no data

The median follow-up time for the total cohort of 119 patients was 15.1 months (m) (range 0.2–99 m). Median survival of the entire group was 13.8 m (95% CI 9.6–20). There was an improved survival rate in patients with good PS, epithelioid subtype histology and patients with response to chemotherapy. Patients with epithelioid subtype presented a median survival of 16.8 m versus 0.8 m sarcomatoid and 13.2 m no other specify (p<0.001). Median survival for patients with PS 0, 1, 2 and 3 was 26.7, 16.5, 2.5 and 0.9 months respectively (p<0.001). Also we found significant differences in survival according to response to chemotherapy. Patients with partial response had a survival of 26.2m vs 17.5 m patients with stable disease and 7.8 m patients with progressive disease (p = 0.003). Stage III-IV and patients older than 75 were associated with worsened survival (OS 13.2 months in stage III-IV and 7.9 months in patients >75 years (p>0.05 in both cases). We did not found differences in survival according to gender, smoking, asbestos exposure and tumor localization (right or left).

### IHC results

**Fig 1:**
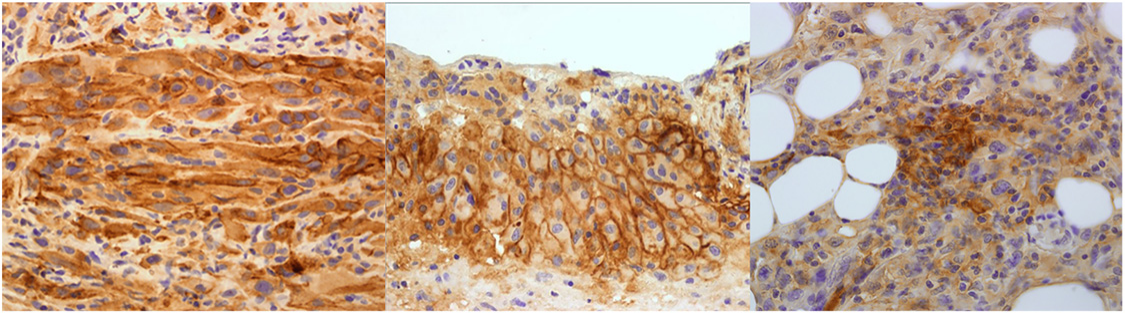
Representatives examples of Hematoxylin eosin immunohistochemical PD-L1 staining in cytoplasm, membrane and inflammatory infiltrate

Of the initial cohort of 119 patients the immunohistochemical analysis for PD-L1 was available in the FFPE of 77 patients. Among the 77 MPM samples examined in our study 16 (20.8%) expressed PD-L1 and 61 (79.2%) were negative. Overall, all of these positive cases displayed cytoplasmic staining and in 14 cases the localization was in membrane. In addition expression was also detected in infiltrating lymphocytes in 4 patients, and all of these patients were also stained in membrane and cytoplasm (Fig. 1).

**Fig 2:**
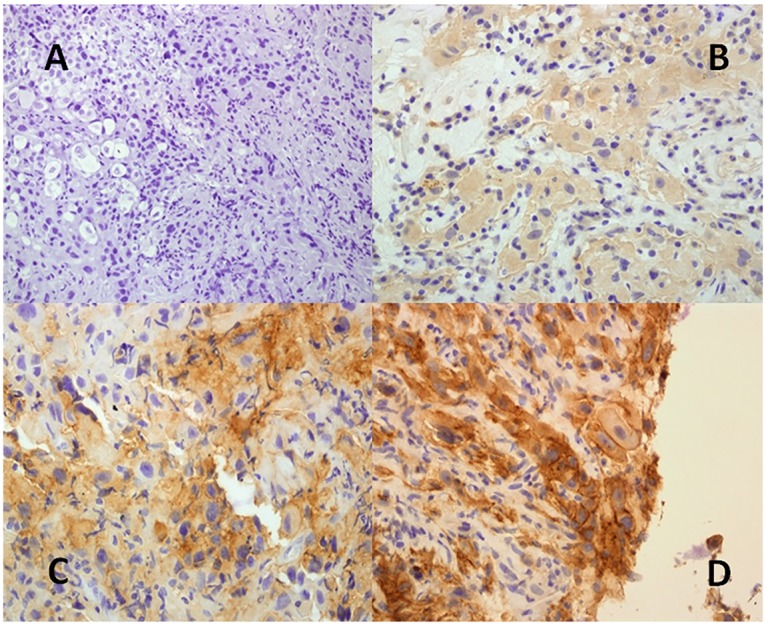
Intensity of PD-L1 staining. A: IHC negative; B: weak expression; C: moderate expression; D: strong expression

The percentage of tumor cells was low in the majority of positive cases. We found 10 (62.5%) PD-L1 positive patients had <5% tumor cells, 3 patients (18.7%) 5–10% tumor cells and 3 patients (18.7%) more than 10% of tumor cells. Analyzing the intensity of staining from the 16 positive patients PD-L1 was weak in 9 patients (56.2%), moderate in 4 patients (25%) and strong in 3 patients (18.8%) (Fig. 2).

We evaluated the presence of TIL and we observed all patients had TIL in the tumors without a predominant immune infiltrate CD4 or CD8. No association was found between expression of PD-L1 and TIL (p = 0.075). In the PD-L1 positive patients we detected an increased infiltration of TILs above average in 61% of patients for CD8+ and in 53% of patients for CD4+.

The univariate relationship between clinical variables and PD-L1 was investigated and a significant correlation between the PD-L1 expression and histology was found (Table 2). We detected that in the group of patients with no epithelioid histology there was more PD-L1 positive than in the epithelioid histology group (no epithelioid positives 9 of 24 patients (37.5%) and epithelioid group positive 7 of 53 patients (13.2%), p = 0.033). The positive patients of the no epithelioid group comprised 5/5 (100%) biphasic, 2/4 (50%) sarcomatoid and 2/11 (18%) histology type not specified of MPM.

Expression of PD-L1 in the tumor by either cytoplasmic, membrane or immune infiltrating cells did not correlate with patient’s gender, asbestos exposure, clinical stage, chemotherapy regimen, response to treatment or TILs.

**Table 2:** Patients characteristics according PD-L1 expression.

Characteristic	PD-L1 + (n,%)	PD-L1 - (n,%)	P
Median age	69	66	0.6
Sex			0.38
Male	11 (68)	43 (70)	
Female	5 (32)	18 (30)	
Histology			0.003
Epithelial	7 (43.7)	46 (77.7)	
No epithelial	9 (56.2)	15 (28.3)	
Smoker	10 (63)	33 (54)	0.61
Asbestos exposure	5 (32)	31 (51)	0.14
Stage III-IV	13 (81)	48 (79)	0.26
Chemotherapy	10 (63)	45 (74)	0.3

### Status of PD-L1 expression and relationship with survival

**Fig 3:**
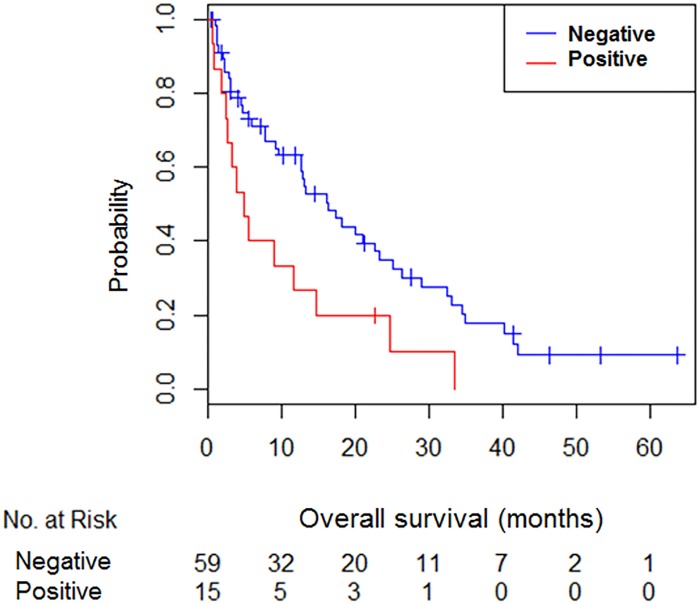
Kaplan-Meier overall survival according to PD-L1 expression

In our series, PD-L1 expression was associated with outcomes. Patients with PD-L1 positive expression presented shorter survival than PD-L1 negative patients (Fig. 3). Median survival for PD-L1 positive patients was 4.8 months and 16.3 months for PD-L1 negative patients (p = 0.012).

Additionally, we analyzed the outcomes for survival according to the intensity of staining and the percentage of positive tumor cells. Median survival for patients with 1–5%, 5–10% and more 10% tumor cell stained was 4.8, 5.6 and 2.6 months respectively (p = 0.9). Median survival for patients with weak, moderate and strong PD-L1 staining was 6.1, 5.2 and 2.6 months respectively (p = 0.9). However, this was an exploratory analysis with a small number of patients and the interpretation of the results must be considered carefully.

Using multivariate analysis with a Cox regression model that included significant variables in the univariate model, we found that PD-L1 remain significant prognostic factor for survival (HR 2.08, 95% CI 1.12–3.88; p = 0.021).

## Discussion

The aim of this study is to investigate the baseline expression of PD-L1 in patients with advanced MPM and to correlate the expression with the outcome. Our results show that MPM express PD-L1 and is associated with poor prognostic.

PD-L1 regulates the cellular immune response and has been shown to be expressed in different tumors, including glioblastoma, pancreas, ovarian, breast, renal, head and neck, esophageal and non-small cell lung cancer (NSCLC) [16–21,23]. Recently, clinical trials using human antibodies directed against critical immune checkpoint molecules have shown promising antitumor activity in several malignancies. Two phase I clinical trials targeting the PD-1/PD-L1 signaling pathway in patients with advanced solid tumors, the majority of them were heavily pretreated, have reported objectives responses between 18–28% of patients [23,24]. In a subset of 42 patients with tumor sample available for assessment PD-L1 expression on the surface of tumor cells with 5H1 antibody, the PD-L1 expression was associated with improved outcome following antiPD1 therapy [23]. A subsequent study in melanoma with this drug showed that although PD-L1 does correlate with response, PD-L1 negative patients can respond to nivolumab [30]. Since these publications other authors have evaluated the association of PD-L1/PD1 therapies with responses [24,25,31].

We found that PD-L1 is expressed in 20% of MPM and no epithelioid subtype expresses PD-L1 more frequently than epithelioid. In this study we evaluate a novel antibody (E1L3N) using a threshold of 1% positive staining of malignant cells to determine whether the tumors were scored as positive or negative for PD-L1. With this threshold, an association between cases scored as positive for PD-L1 expression and clinical responsive to PD-L1 has been reported with the MPDL3280A and 2CC3 antibodies [25,26]. However thresholds for positivity have not been clearly defined for all the PD-L1 antibodies and definition of “positive” PD-L1 expression is variable across studies. In the study of Topalian staining with 5H1 in membrane with a cutoff of 5% tumor cells stained was considered PD-L1 positive [23]. A posterior study with this drug showed that many patients with PD-L1 negative tumors can respond to PD-1 blockade [30]. The 2CC3 assay defined PD-L1 positivity tumor surface expression >1%. However variations in this assay and its application have emerged across tumor types. In NSCLC immune and tumor cells were included in the cutoff and a strong expression (staining ≥50%) derived greater clinical benefit than patients with weak or negative score PD-L1 expression [31]. In head and neck with the same antibody only PD-L1 expression on tumor cells was considered [32].

PD-L1 expression is measured most commonly by IHC however no test is uniformly accepted as the standard for quantitating PD-L1 expression. Multiple, distinct, companion assays for PD-L1 positivity have been developed, but there is not yet comparison, standardization, or prospective validation of these assays. Thus it is hard to determine whether there is consistency in the tumors that are declared to be PD-L1 positive. Additionally a different and more reproducible methodology for evaluating PD-L1 has been evaluated with the measurement of mRNA in breast cancer and lung cancer showing association with better outcomes [33,34].

Previous studies have shown that PD-L1 can be expressed by multiple components of the tumor microenvironment, including tumor cells themselves and infiltrating immune cells. The biological consequences of PD-L1 expression depend on cell membrane localization and cytoplasmic staining may represent intracellular stores of PD-L1 which may be developed to the cell surface depending on appropriate stimulation [35]. However the clinical significance of localization of PD-L1 is not known and the different antibodies used in the clinical trials focus on different localizations. The clinical trial with MPDL3280A suggests that tumor-expressed membrane PD-L1 and immune infiltrate cell correlates with response to anti-PD-1 therapy [25]. In a recent report 5H1 was used to evaluate PD-L1 expression in a series of 106 MPM patients founding that PD-L1 was expressed in 42 patients (40%). In this study most cases expression was cytoplasmic (18 patients, 43%), in many cases there was cytoplasmic and membranous staining (14 patients, 33%) and exclusive membranous staining was less common (10 patients, 24%). In our series all patients presented cytoplasmic and majority of them membrane staining of PD-L1.

An association of PD-L1 expression with histology has been reported by other authors. Mansfield, in the series of MPM reported that every case of MPM with sarcomatoid differentiation expressed PD-L1 [28]. In a study of lung cancer sarcomatoid differentiation express PD-L1 in 69.2% of patients using 5H1 antibody [36]. Our observations are consistent with these data in MPM using a different antibody showing an association of PD-L1 protein expression with no epithelioid histology. Association PD-L1 with histology also has been reported in breast cancer. Basal subtype cell lines have higher constitutive expression levels compared to luminal cell lines [37]. Among other characteristics analyzed in our study (gender, asbestos exposure, clinical stage, schema of chemotherapy, response to treatment or TIL) we did not observe any association with PD-L1 expression.

A strong correlation between PD-L1 expression on tumor cells and prognosis has been observed in some cancer [16–19,22]. Our first finding is that PD-L1 was expressed in MPM more frequently in no epitheliod patients and PD-L1 negative patients had a significantly better prognosis than the positive patients. The effect of PD-L1 status on prognosis was indistinctive of the histology. Compared with epithelioid, no epithelioid tumors are more aggressive and cytotoxic chemotherapy is generally ineffective [38,39]. In the study of MPM published by Mansfield PD-L1 expression was associated with worse prognosis. PD-L1 status may be a critical factor to promote tumor growth and metastasis in MPM. However we can not exclude other additional factors which could influence the prognosis. Some reports have shown that PI3K pathway is responsible of PD-L1 activation and PI3K has been associated with poor prognosis in MPM [21,40]. EGFR expression assessed by immunohistochemistry has been related with prognosis in some reports [41]

On the basis of the current status of MPM, immunotherapy has also been considered as one of the novel therapeutic approaches. Recently, tumor-specific immunotherapy using vaccination with antigen peptides of tumor associated antigens (TAA) has been conducted although the number of patients is small. Mesothelin is an immunogenic glycoprotein highly expressed in MPM. In preclinical studies and two phase I trials targeting mesothelin showed clinical activity [42–44]. Wilms tumor-1 (WT-1) is highly expressed in MPM and phase I peptide vaccination elicited T-cell response [45]. An uncontrolled phase II study with tremelimumab in 29 patients who progressed to chemotherapy a 31% of disease control rate was found and almost 40% of participants were alive at two years [46]. In a tumor model of mesothelioma they reported PD-L1 is ubiquitously expressed in the tumor stroma, and that PD-L1 blockade results in T cell activation [27]

In summary, this is the first report measuring PD-L1 protein levels in MPM with the E1L3N antibody. Our data suggest association between the presence of PD-L1 with histology and survival. The measurement of PD-L1 has the potential to identify subsets of MPM and also may predict for response to PD-1/PD-L1 pathway blocked. We suggest that PD-L1 expression in MPM is a candidate molecular marker that warrants further exploration for use in selecting MPM patients for immunotherapy.
